# A Rare Initial Presentation of Gastric Cancer with Multiple Osteolytic Lesions

**DOI:** 10.1155/2015/689431

**Published:** 2015-04-23

**Authors:** Asad Jehangir, Kim Aderhold, Priya Rajagopalan, Oluwaseun Shogbesan, Sharon Swierczynski, Anam Qureshi, Qasim Jehangir, Christian Espana Schmidt

**Affiliations:** ^1^Department of Internal Medicine, Reading Health System, Spruce Street/6th Avenue, West Reading, PA 19610, USA; ^2^Department of Pathology, Reading Health System, Spruce Street/6th Avenue, West Reading, PA 19610, USA; ^3^King Edward Medical University, Mayo Hospital Road, Nelagumbad, Anarkali, Lahore 54000, Pakistan; ^4^Rawalpindi Medical College, Tipu Road, Rawalpindi 46000, Pakistan; ^5^Reading Health System, Spruce Street/6th Avenue, West Reading, PA 19610, USA

## Abstract

Gastric cancer is the 12th leading cause of cancer-related deaths in the United States and commonly metastasizes to the bones. However, the presentation of gastric cancer as bony metastases without preceding gastrointestinal symptoms is rare which has been infrequently reported in the literature. Moreover, leptomeningeal carcinomatosis is an unusual complication of gastric cancer accounting for less than 1 percent of these patients. We present a unique case of a middle aged male who presented to the emergency department with worsening backache which started one month priorly. The only abnormal laboratory test was an elevated alkaline phosphatase of 154 IU/L. The imaging of his spine showed osteolytic lesions which on biopsy revealed signet ring cells. A small 2 cm ulcerated mass was found on esophagogastroduodenoscopy at the gastric cardia which on biopsy revealed signet ring gastric carcinoma. The patient received chemotherapy with capecitabine and oxaliplatin as well as radiation and showed a good response initially. A few months later, he presented with persistent worsening headaches and on brain imaging was found to have leptomeningeal carcinomatosis. Ten months after the diagnosis of gastric carcinoma, he passed away.

## 1. Introduction

Fifty percent of gastric adenocarcinomas can initially present with distant metastasis. Disseminated bony metastases as the first clinical manifestation are seen in exceptional cases and generally present in the later stages of an established disease. Furthermore, leptomeningeal carcinomatosis (LMC) is a rare complication of gastric cancer seen in only about 0.16 to 0.69% of cases [1]. We describe an unusual presentation of gastric cancer with multiple osteolytic metastases complicated by LMC few months after the diagnosis despite chemotherapy.

## 2. Case

A 48-year-old male with history of mild gastroesophageal reflux disease presented to the emergency with a one-month history of worsening low back pain without a preceding trauma. The pain had gradually increased to 10/10 in severity and impaired his ambulation over the previous 2 days. He denied any lower extremity pain, weakness, numbness or tingling, or fecal or urinary incontinence. He also reported right chest discomfort that began with a “painful crack” while he was bowling about a week before. His backache and chest pain did not improve with a brief course of NSAIDs. He denied nausea, vomiting, hematochezia, melena, or weight loss. His family history was negative for any malignancies.

**Figure 1:**
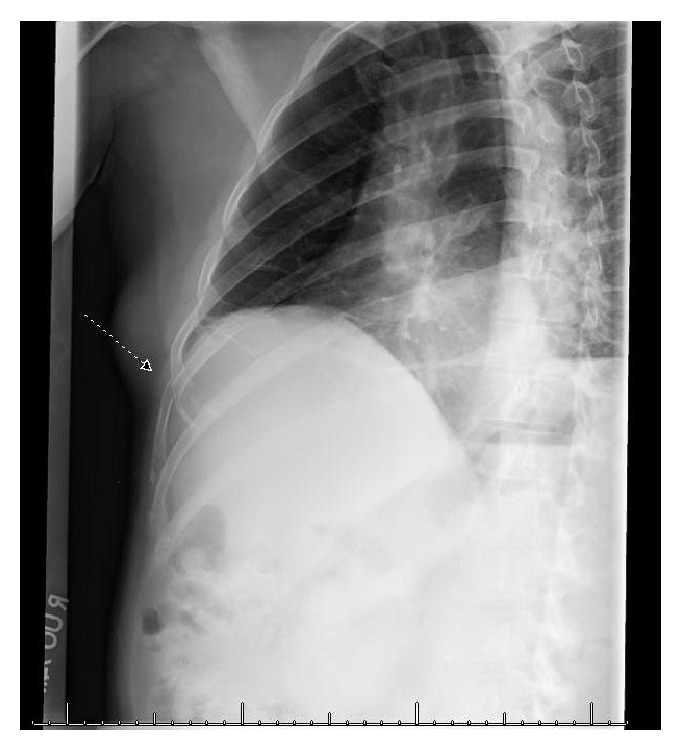
X-ray of the right sided ribs revealing a fracture involving the lateral aspect of the 7th rib.

**Figure 2:**
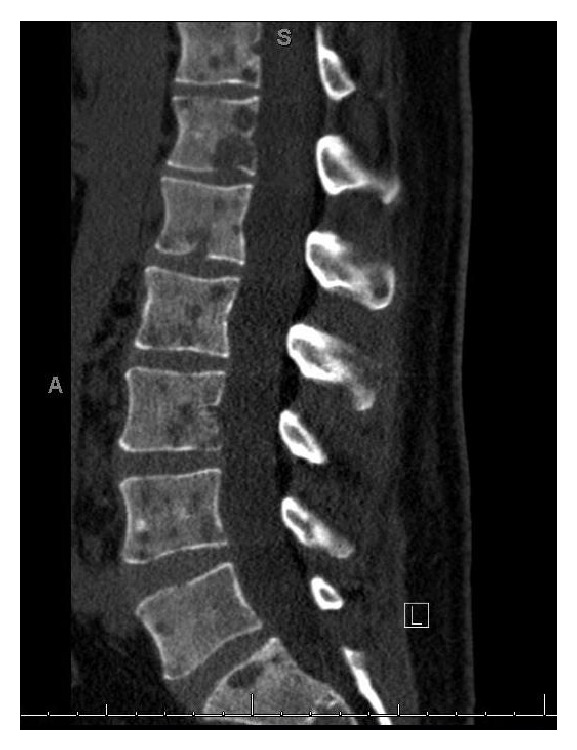
CT lumbar spine without contrast showing innumerable lytic lesions seen throughout the lumbar spine.

**Figure 3:**
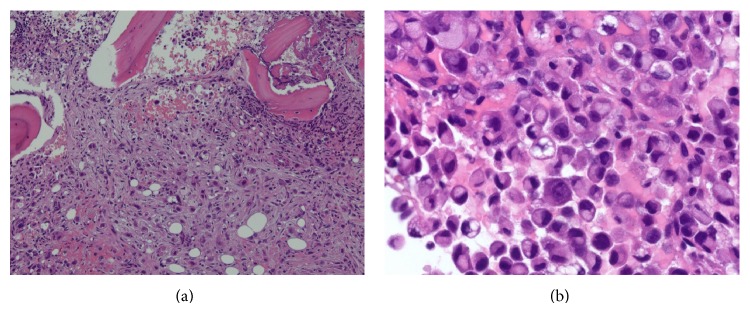
(a) Metastasis to the lumbar spine. Histologic sections of a core biopsy of the L3 vertebral body demonstrate near complete marrow replacement by a proliferation of malignant cells with associated fibrosis ((a), 10x). (b) Metastasis to the lumbar spine. On high power (40x), the infiltrate is comprised of signet ring cells morphologically identical to the gastric cardia biopsy (see Figure 4(b)).

**Figure 4:**
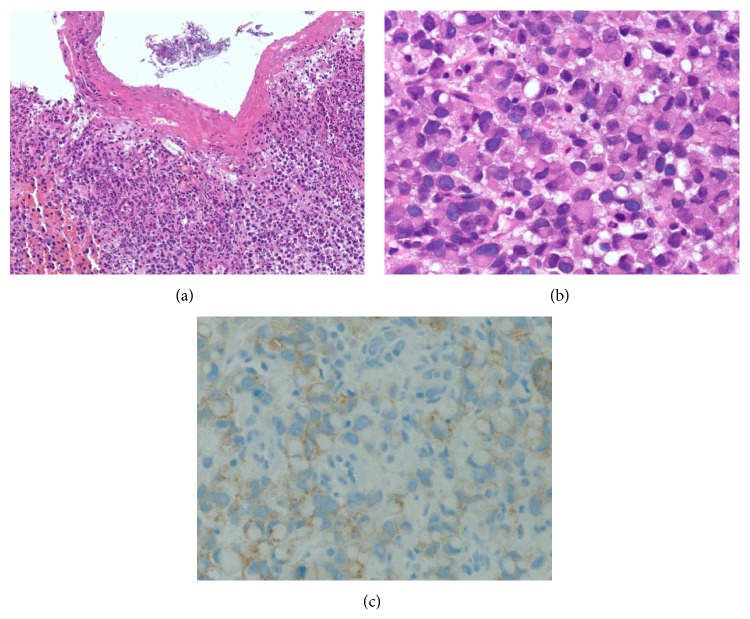
(a) Poorly differentiated adenocarcinoma of the gastric cardia. Histologic sections demonstrate erosion of the surface epithelium with diffuse involvement of the lamina propria by an atypical infiltrate comprised predominantly of discohesive cells ((a), 10x). (b) Poorly differentiated adenocarcinoma of the gastric cardia. On high power (40x), the cells in the infiltrate demonstrate prominent signet ring cell morphology, diagnostic of poorly differentiated adenocarcinoma. (c) Poorly differentiated adenocarcinoma of the gastric cardia. An immunohistochemical stain for HER2 interpreted as being equivocal ((c), 40x).

On examination the patient did not have any spinal tenderness or signs of spinal cord compression and had normal strength, sensation, and reflexes in the lower extremities. The only significant abnormality on the laboratory tests was an elevated alkaline phosphatase of 154 IU/L (reference range 38–110 IU/L). Other laboratory values including calcium (9.9 MG/DL), creatinine (0.94 MG/DL), hemoglobin (15.6 G/DL), platelet count (320 CMM), and PSA (0.45 NG/ML) were all normal. An X-ray of the chest showed a fracture of the lateral aspect of the right 7th rib (Figure 1). Computerized tomography (CT) of the spine revealed several lytic lesions throughout most of his spine (Figure 2) which initially raised suspicion for multiple myeloma; however the findings of normal calcium, creatinine, and protein levels prompted evaluation for an alternative diagnosis. A CT chest did not reveal any lung or thyroid masses. A left transpedicular core biopsy of the L3 vertebra was performed which surprisingly was consistent with adenocarcinoma with signet cell features (Figures 3(a) and 3(b)). A CT of the abdomen and pelvis showed multiple prominent retroperitoneal lymph nodes which were not pathologically enlarged; however, their abnormal number was suspicious for early metastatic disease. Esophagogastroduodenoscopy revealed a small 2 cm ulcerated mass at the gastric cardia which was not visualized on the CT scan. A biopsy indicated invasive poorly differentiated gastric adenocarcinoma with signet ring cells (Figures 4(a) and 4(b)). An immunohistochemical stain for human epidermal growth factor receptor 2 (HER2) was equivocal (Figure 4(c)). HER2 fluorescence in situ hybridization analysis was performed which did not reveal any evidence of HER2 gene amplification.

**Figure 5:**
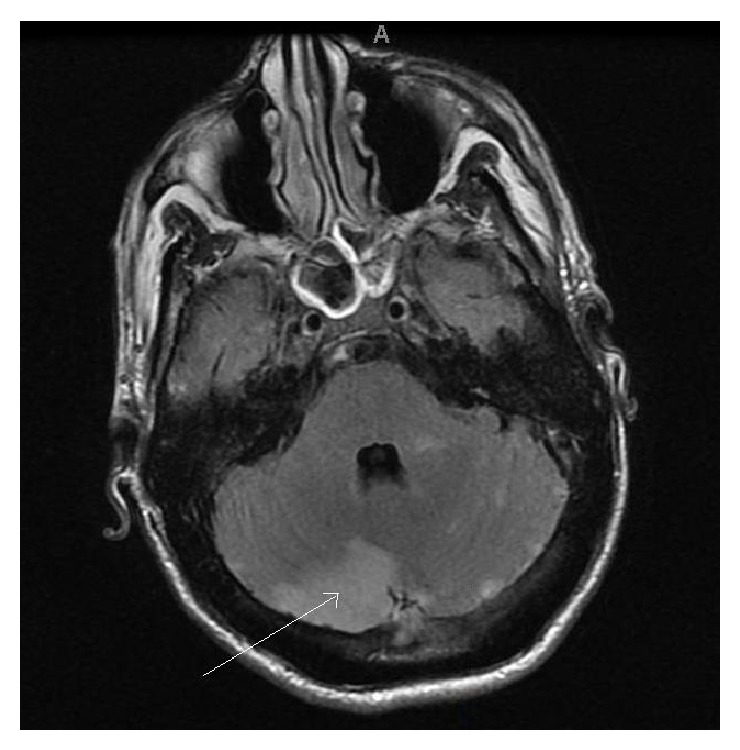
MRI brain with and without contrast revealing multiple foci of predominantly cortical signal change with the largest area involving the medial right cerebellar hemisphere (white arrow). The appearance is most compatible with subacute infarcts, presumably from an embolic source. There are no enhancing masses to suggest metastatic disease.

The patient underwent chemotherapy with capecitabine and oxaliplatin along with radiation with good initial response; however, after a few months he reported persistent headaches. Head imaging demonstrated leptomeningeal carcinomatosis. Ten months after the initial diagnosis of metastatic gastric cancer, he was brought to the emergency department after multiple episodes of loss of consciousness. A brain MRI revealed acute infarcts in multiple vascular territories (Figure 5), presumably caused by a hypercoagulable state secondary to malignancy. The patient's condition continued to deteriorate, and he expired approximately two weeks later.

## 3. Discussion

Gastric cancer is the 12th leading cause of cancer-related death in the United States and accounted for approximately 11,000 deaths nationwide in 2014 [2]. There has been a progressive decline in the incidence of intestinal type gastric cancer; on the contrary, the diffuse type of gastric carcinoma is being diagnosed more frequently, particularly the signet ring cell type, which constitutes 3.4 to 29% of all cases of gastric carcinomas [3, 4]. The diffuse type often presents in an advanced stage and at a younger age [4]. Most cases (99%) of signet ring cell carcinoma originate in the stomach [4].

Bone metastases from gastric cancer, more commonly seen in signet ring cell type, portend a poor prognosis. The reported incidence of bones metastases varies widely from as low as 1% in clinical practice to as high as 45% in screening studies for bone metastases, implying that many cases are asymptomatic [5]. Bone metastases are usually detected after a diagnosis of gastric cancer has already been established. Gastric cancer presenting as bone metastases without any preceding gastrointestinal symptoms has been infrequently reported in the literature [6, 7]. Such rare cases are generally associated with significantly larger primary gastric lesions in contrast to the small 2 cm mass of the gastric cardia seen in our patient. The most likely route of spread from a primary gastric lesion to the bone is via lymphatic channels, although dissemination may also occur through portal and nonportal veins [8]. The most common sites of involvement reported in the literature include spine, ribs, scapula, and pelvis [9]. Patients with bone metastases may present with refractory pain or pathological fractures. Various laboratory abnormalities that suggest the possibility of bone metastases include elevated alkaline phosphatase (ALP), increased LDH, and anemia or thrombocytopenia. Of interest, the only deranged laboratory finding in our patient was elevated ALP [8, 10].

Our patient also developed leptomeningeal carcinomatosis which is usually seen as a complication of hematological malignancies and manifests less commonly in solid malignancies [11]. Melanoma and lung and breast cancer are the more common solid tumors associated with LMC [11]. LMC resulting from an underlying gastric malignancy has been rarely reported in the literature and is associated with poor outcomes. It commonly manifests as headaches; other neurological manifestations may include weakness, dizziness, altered mental status, or seizures [12]. There is no single test sensitive enough to establish the diagnosis, although a combination of CSF cytology and MRI imaging can assist with a combined sensitivity of over 90% [13].

Chemotherapy for the treatment of advanced metastatic gastric carcinomas may be associated with better quality of life than symptomatic treatment alone. Intrathecal chemotherapy may be used in cases of LMC [11, 14]. Adjuvant trastuzumab may also be utilized for HER2 positive lesions [14]. However, chemotherapy is not advised for patients with Eastern Cooperative Oncology Group score of 3 or higher and must be carefully utilized in a selective group of patients after extensive counseling. Those patients not responding should be recognized early [9]. Advanced signet ring cell carcinoma carries a poor prognosis compared to other types of gastric cancer [4]. Other factors associated with poor outcomes include microinvasion of vessels and tumor location; tumors involving the whole stomach understandably have a worse prognosis compared to lesions localized in the antrum and the body [4]. Mean survival time is 4-5 months with bone metastases from gastric cancer and even less about 3 months with LMC [10, 15, 16].

## 4. Conclusion

Multiple rounded osteolytic lesions and pathologic fractures are often presumed to be multiple myeloma. Also included in the differential are metastases from solid organs like prostate, breast, lung, thyroid, or kidney. However, it is prudent to consider gastric cancer as the possible primary site of disease even in the absence of overt gastrointestinal symptoms. Leptomeningeal carcinomatosis is a rare but invariably fatal complication of gastric cancer that frequently manifests as headaches; however, other neurological complaints may also be present.
